# Exploring the Involvement of New Members of the Interleukin Family in Cardiovascular Disease

**DOI:** 10.2174/011573403X330079241213071055

**Published:** 2025-01-21

**Authors:** Abdullah Al Noman, Sanzida Alam Flora, Monty Datta, Fahmida Afrose, Nushaiba Binte Hasan, Tahamina Akhter, Nayeema Jameel Anuva, Rashmi Pathak, Himanshu Sharma

**Affiliations:** 1 School of Pharmacy, BRAC University, Dhaka 1212, Bangladesh;; 2 Department of Zoology, Government Tolaram College, National University of Bangladesh, Gazipur 1704, Bangladesh;; 3 Department of Pharmacy, Varendra University, Rajshahi 6204, Bangladesh;; 4 Department of Pharmacy, Invertis University, Bareilly (UP)- 243123, India;; 5 Department of Pharmacy, Teerthanker Mahaveer College of Pharmacy, Teerthanker Mahaveer University, Moradabad (UP)- 244001, India

**Keywords:** Cardiovascular disease, interleukin, IL-27, IL-31, IL-32, IL-33, IL-28, IL-29, atherosclerosis, heart failure

## Abstract

Cardiovascular diseases remain a significant reason for illness and death globally. Although certain interleukins have been extensively researched about cardiovascular disease (CVD), new findings have identified unique members of the interleukin family that could potentially play a role in cardiovascular well-being and ailments. This review discusses the current understanding of the role of these recently identified interleukins, such as IL-27, IL-31, IL-32, IL-33, and the IL-28 group (IL-28A, IL-28B, IL-29), in the development of cardiovascular diseases. Every interleukin has various impacts achieved through particular receptors and signaling pathways that affect inflammatory processes, differentiation of immune cells, and the functioning of blood vessels. IL-27 controls the development of inflammatory Th17 cells and might decrease inflammation in atherosclerosis. IL-31 plays a role in the interaction between the immune system and nerves, as well as in itching. IL-32 enhances the generation of inflammatory proteins and has been linked to coronary artery disease. IL-33 has beneficial effects on the cardiovascular system, whereas its imitation receptor sST2 could potentially be used as a biomarker. Additional studies are needed to investigate the antiviral and immune-system regulating effects of the IL-28 group in cardiovascular diseases. In general, explaining the ways in which new interleukins contribute to the progression of cardiovascular diseases can help discover fresh targets for therapy and new approaches toward enhancing the prevention and treatment of heart disorders. Additional research on the way these cytokines engage with established disease pathways is necessary.

## 1 INTRODUCTION

Cardiovascular diseases (CVD) continue to pose a serious threat to the world's health, imposing an enormous impact on morbidity and death. It has taken a lot of research to identify the complex mechanisms behind CVD and create efficient treatment plans [1]. It is essential to grasp the complex molecular pathophysiological processes at play. In recent years, there has been a growing recognition of the role played by various pro-inflammatory cytokines in the development and progression of several cardiovascular diseases (CVDs), marking them as states characterized by pro-inflammation. This emphasizes how important it is to learn more about the precise functions of these cytokines in cardiovascular disease and health [2]. Previous studies have indicated that new members of the interleukin family (IL-27, IL-28, IL-29, IL-31, IL-32) have a wide range of immunosuppressive characteristics and are important in a number of inflammatory diseases [3-7]. The interleukin (IL) family of proteins has come under more investigation recently for their potential significance in cardiovascular health and illness. Interleukins, an array of signaling molecules, are essential for controlling inflammatory processes and immunological reactions. Recent developments have shown the existence of novel interleukins with potential consequences in cardiovascular disease, even though several members of the IL family have been thoroughly researched in the context of CVD [1]. Promising new study directions in the area of cardiovascular immunology have been made possible by the identification of these novel interleukins. Finding out more about their functions and mechanisms in CVD can help us understand the complex interactions between immune system dysregulation, inflammation, and vascular function. Atherosclerosis, myocardial infarction, heart failure, and hypertension are just a few of the cardiovascular disorders that these recently discovered interleukins have the potential to treat [8]. Understanding their roles in the pathophysiology of disease can help in the creation of novel therapeutic approaches that could successfully control immune responses, reduce inflammation, and reduce the risk of cardiovascular disease. However, there are challenges in bridging the gap between clinical application and laboratory research, including in vitro and animal investigations. The importance of large-scale clinical trials is still largely unknown, despite evidence tying them to increased cardiovascular risk [9]. We intend to explore the state of knowledge on the contribution of these recently identified interleukins to cardiovascular disease in this thorough study. This review ultimately intends to increase scientific understanding and stimulate additional research into the complex interactions between interleukins and cardiovascular diseases, with the end objective of improving patient outcomes and lowering the worldwide burden of CVD.

## 2 BRIEF DISCUSSION ON THE ROLE OF INFLAMMATION IN DISEASES

The immune system triggers a response to protect our body from harmful stimuli, which is known as inflammation. It can be of two types: acute and chronic [10]. Acute inflammation is favourable to the body and triggers a response to traumatic tissue injury and pathogens [10, 11]. Chronic inflammation can be acute inflammation when it is uncontrollable, which leads to diseases [10]. However, tissue injury or pathogens turn on chemical signals to activate leukocytes and help in chemotaxis to go to damaged areas. The activated leukocytes help in producing various immune cells, including cytokines, mast cells, macrophages, dendritic cells, histamine bradykinin and many more, which play roles in the immune system. However, chronic inflammation has different pathways due to many conditions [12]. That is the reason it can lead to additional long-term illnesses that ultimately lead to demise. Several factors are connected to chronic inflammation disease, including age, eating habits, being overweight, tobacco use, reduced levels of reproductive hormones, anxiety, and sleep problems; numerous additional factors have also been identified. IL-4, IL-5, IL-6, and IL-7 cytokines stimulate the humoral reaction, while IL-1, IL-2, IL-3, IL-4, IL-7, IL-9, IL-10, IL-12, interferons, and TNFα and β elicit the cellular response during chronic inflammation [13]. In the world, 3 of 5 people die because of chronic inflammation disease. Some chronic inflammation diseases are diabetes, cardiovascular disease (CVD), stroke, cancer, arthritis, and many more. According to the American Diabetes Association survey result, 9.4% of American people had diabetes in 2015, 1 of the 3 deaths due to CVD and 20 deaths due to stroke in 2017, 20% of people affected by arthritis in 2020, 8.4% of children and 8.2% adults affected by hay fever in 2015, 6.4% affected with Chronic Obstructive Pulmonary Disease (COPD) in 2014; in the United States [14]. An important factor in the development, course, and symptoms of CVD is inflammation. Targeting inflammation may provide a unique strategy for lowering the risk for acute CV events, despite the fact that safely controlling inflammation with targeted therapies remains a challenge, as seen by the outcomes of recent prospective studies. Scientists have discovered that statin drugs, which have anti-inflammatory qualities, may be able to lessen atherosclerosis in people with cardiovascular disease, even though it is not shown that inflammation causes cardiovascular disease [15].

## 3 CYTOKINE FAMILY IN CVD

According to a study, people who had their levels of interleukin-6 (IL-6) less than 1.65 ng/L gained the greatest advantages from treatment for cardiovascular illness. It is important to comprehend the distinction between IL-6 and IL-6R disruption because IL-6 signaling begins when the circulating IL-6 ligand attaches to the soluble or membrane-bound IL-6R. This is in comparison to the process of transmitting signals through the IL-6R attached to the membrane (classic signaling), which is commonly considered to have an anti-inflammatory effect. The transmission of signals through soluble IL-6R (trans-signaling) is generally believed to be inflammatory. Those with higher levels of insoluble IL-6R polymorphisms are associated with a lower risk of CAD. Our findings support the advantages of disrupting IL-6 signaling in coronary artery disease (CAD) by inhibiting either IL-6 or IL-6R, which is consistent with other research on IL-6R variations. By using this technique, the risk of atrial fibrillation and CAD development can be reduced [15, 16]. Studies conducted in cohorts whose primary goal is to avoid cardiovascular disease have discovered a distinct relationship between blood levels of CRP and IL-6 and the risk of developing CVD. Moreover, in the CANTOS study, the degree of IL-6 reduction attained was positively correlated with the rate of cardiovascular risk reduction. These results add to the existing epidemiological and experimental evidence in this field. IL-6 helps to increase the presence of cellular adhesion molecules in the walls of arteries, raises the permeability of blood vessels, damages the function of the lining of blood vessels, and boosts the growth of smooth muscle in blood vessels. All of these actions are involved in the relationship between the dosage of IL-6 and its effects. As a result, larger reductions in IL-6 signaling, evaluated by a decrease in hs-CRP, would be linked to greater advantages in reducing the risk of cardiovascular ailments. Chronic inflammation has emerged as a recent focus for treating cardiovascular disease (CVD). The gathered evidence emphasizes the significance of interleukin (IL)-6 in atherosclerosis and has sparked curiosity about medications that impact IL-6 signaling as a potential treatment option. Based on findings from the Canakinumab Anti-Inflammatory Thrombosis Outcomes Study (CANTOS) study, reducing the IL-6 and hs-CRP levels was observed alongside the cardiovascular impact of IL-1 inhibition using canakinumab. The overall decrease in hs-CRP levels is likely equal to the impact of preventing IL-6 signaling in lowering cardiovascular disease risk. As a result, individuals who have the greatest initial levels of hs-CRP should be the main focus for reducing the risk of cardiovascular disease by inhibiting IL-6 signaling [16]. When hematopoietic stem cells proliferate clonally due to acquired leukemic mutations in genes like DNMT3A or TET2, this phenomenon is known as clonal hematopoiesis of undetermined potential (CHIP). Myocardial infarction is often linked to CHIP in humans. We hypothesized that antagonizing the IL-6 pathway in CHIP carriers will reduce their risk of cardiovascular disease (CVD) since CHIP speeds up atherosclerosis and elevates IL-6/IL-1 expression in mice [17]. Based on the findings, IL-17A might serve as a useful indicator for heart-related illnesses, as individuals with lower IL-17A levels in their blood had a higher chance of developing cardiovascular diseases. Although IL-17 has both effects that promote atherosclerosis and effects that protect against it, there are still some aspects of its potential roles in cardiovascular disease that are not well understood. Evidence indicates that IL-17A may have a safeguarding role in certain scenarios, like in individuals experiencing a heart attack, where reduced levels of IL-17A in the bloodstream have been associated with mortality and repeated severe cardiovascular incidents. However, the medication that opposes IL-17A has demonstrated the potential to reduce the likelihood of heart disease in patients [18]. The basic function of the IL-17 cytokine family is to cause inflammation, and it is mainly produced by Th17 cells. This cytokine is very important in the development of certain inflammatory diseases. The biological function of the IL-17 cytokine IL-17A is the highest among the six members of the IL-17 cytokine family (IL-17A to IL-17F). Certain immune system signaling proteins called cytokines, namely IL-17A and IL-17C, might contribute to the vulnerability of atherosclerotic plaques. However, IL-17E has been observed to defend against the formation of these harmful deposits. Although there is a connection between IL-17F and CVD, its role has not been fully investigated. The role of IL-17B and IL-17D in atherosclerosis is not well understood [19]. Clinical results may be greatly improved by targeting pro-inflammatory cytokines that are abundantly expressed in CVD, as shown by the CANTOS experiment. Inhibition of the multifunctional pro-inflammatory cytokine IL-1b, often known as the gatekeeper of inflammation owing to its capacity to control various immunological cascades, was the primary goal of the (CANTOS) study, which used a monoclonal antibody to achieve this goal [20]. Leukocyte trafficking and infiltration into atherosclerotic lesions, foam cell production, and cell maturation inside the plaque are all significantly impacted by the chemokine (-receptor) network. Here, we present recent discoveries on the function of several chemokines and their receptors in atherosclerosis. Biomarker Condition Medical relevance in the clinic: simvastatin dramatically reduces CCL2 levels in hypercholesterolemia, especially in the asymptomatic stage of atherosclerosis (AS). In a study involving FH and CAD, a reduction in CCL2 was seen postprandially following an oral unsaturated lipid load. A higher probability of clinically unfavorable events acting as a predictor of CAD and high prevalence of acute myocardial infarction and unstable angina pectoris indicates the likelihood of restenosis after coronary angioplasty. Also, the effects of an oral unsaturated fat load on CCL3, CCL4, and AS progression in FH were studied. In patients with chronic cytokine 5 stable angina pectoris, a rise in CCL5 levels may be an indicator of future restenosis. Acute unstable coronary syndrome variable that predicts Adverse Clinical Events (ACE) in cases of acute myocardial infarction and unstable angina pectoris tends to be higher. Incidence of C-C motif chemokine ligand 17 in coronary artery disease connected favorably with CAD manifestation and severity. Clinical role of CXCL12 acute myocardial infection AMI and CXCL16 acute coronary syndrome (ACS) are indicators of potential harm in humans. As a result, cytokines may present viable targets for the prevention of CVD. Cytokines are critical in controlling the inflammatory response by changing vasoconstriction and obstructing endothelium-dependent dilation [21].

## 4 INTERLEUKINS IN INFLAMMATION PATHWAY IN CVD

**Fig. (1):**
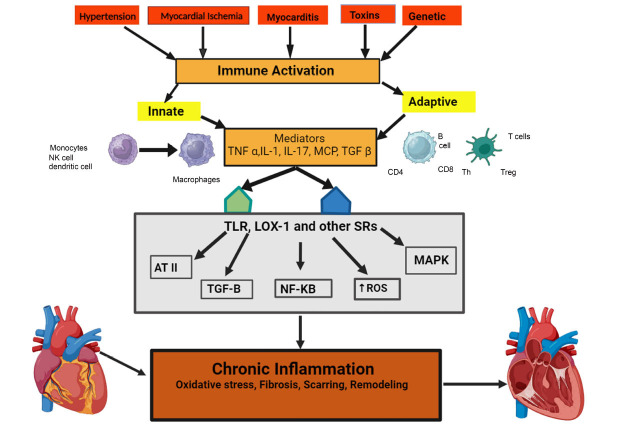
Interleukins in the immune inflammatory system of heart failure.

The classic cytokine that stimulates inflammation is interleukin-1. Interleukins are a crucial modulator of the systemic anti-inflammatory response. IL-1 is a significant inflammatory molecule associated with atherothrombosis since it is situated near the conventional IL-6 signaling cascade. Thus, it has been widely accepted that IL-1 should be the focus of novel vasculoprotective medications. The IL-1 type 1 receptors (IL-1α and IL-1β) are influenced by two stimulants in addition to the naturally occurring IL-1 receptor agonist (IL-1Ra). This agonist competitively prevents IL-1α and β from binding to type 1 receptors. Monocytes, a kind of immune cell, create Pro-IL-1β, which is the inactive early version of IL-1β, at a medically relevant heightened degree of control. Pro-IL-1β requires proteolytic cleavage to become biologically active [22]. IL-1 was previously associated with septic shock as a cardio-depressant factor and nowadays, IL-1 is linked to heart failure (HF) and acute myocardial infection (AMI) in significant pre-clinical and clinical studies. In a large phase III clinical trial, stable patients with prior AMI were protected against recurrent atherothrombotic cardiovascular events by using a monoclonal antibody to inhibit IL-1 activity [23]. Despite advancements in therapy, diagnosis, and prevention strategies, people with heart disease (CVD) still face an elevated risk of recurrent events due to inflammation. Cytokines, which are soluble communication proteins, regulate an inflammatory response to the invasion of the body and damage tissues by impacting the interleukins that trigger the response between cells. The conventional inflammatory protein, interleukin-1 (IL-1), has a key role in the initial response of the body's natural defense system. Increased IL-1 operation has been linked to cardiovascular disease in recent periods and is implicated in the pathogenesis of various inflammatory conditions [23]. Two isoforms of IL-1 are soluble, autocrine, paracrine, and endogenous messenger IL-1β, and membrane-bound, autocrine, as well as paracrine messenger IL-1α. By disinhibiting the I-κB effects of nuclear factor κB, both isoforms increase the inflammatory response by binding the IL-1 receptor (type I) and enlisting the help of adaptor as well as accessory proteins. Alongside IL-1β, another naturally present and soluble receptor inhibitor called IL-1 receptor antagonist (Ra) is formed. Ra attaches to the IL-1 receptor, but in order to bind with IL-1, it activates intracellular signaling pathways. Mainly acting as a warning sign, interleukin-1a initiates the inflammatory series of events, leading to the production of IL-1β, which enhances the inflammatory response even more. An increase in IL-1α levels during the early phase enhances the size of the area affected by the damage caused by lack of blood supply to the heart tissue. On the other hand, IL-1β becomes the main chemical messenger during the later phases, leading to the demise of heart muscle cells, negative changes in the structure of the heart, and, ultimately, heart failure [23]. During hypertension, viral infection and myocardial infection lead to immune activation. The innate and adaptive immune systems are activated by a few factors so that inflammatory responses are triggered and are mediated by cytokine release. Through many receptors along with several pathways, heart is affected and causes heart failure, known as the immune inflammatory system of heart failure (Fig. **1**).

## 5 NEW ADDITION TO THE INTERLEUKIN FAMILY AND THEIR ROLE IN CVD

### 5.1 Interleukin‐27

**Fig. (2):**
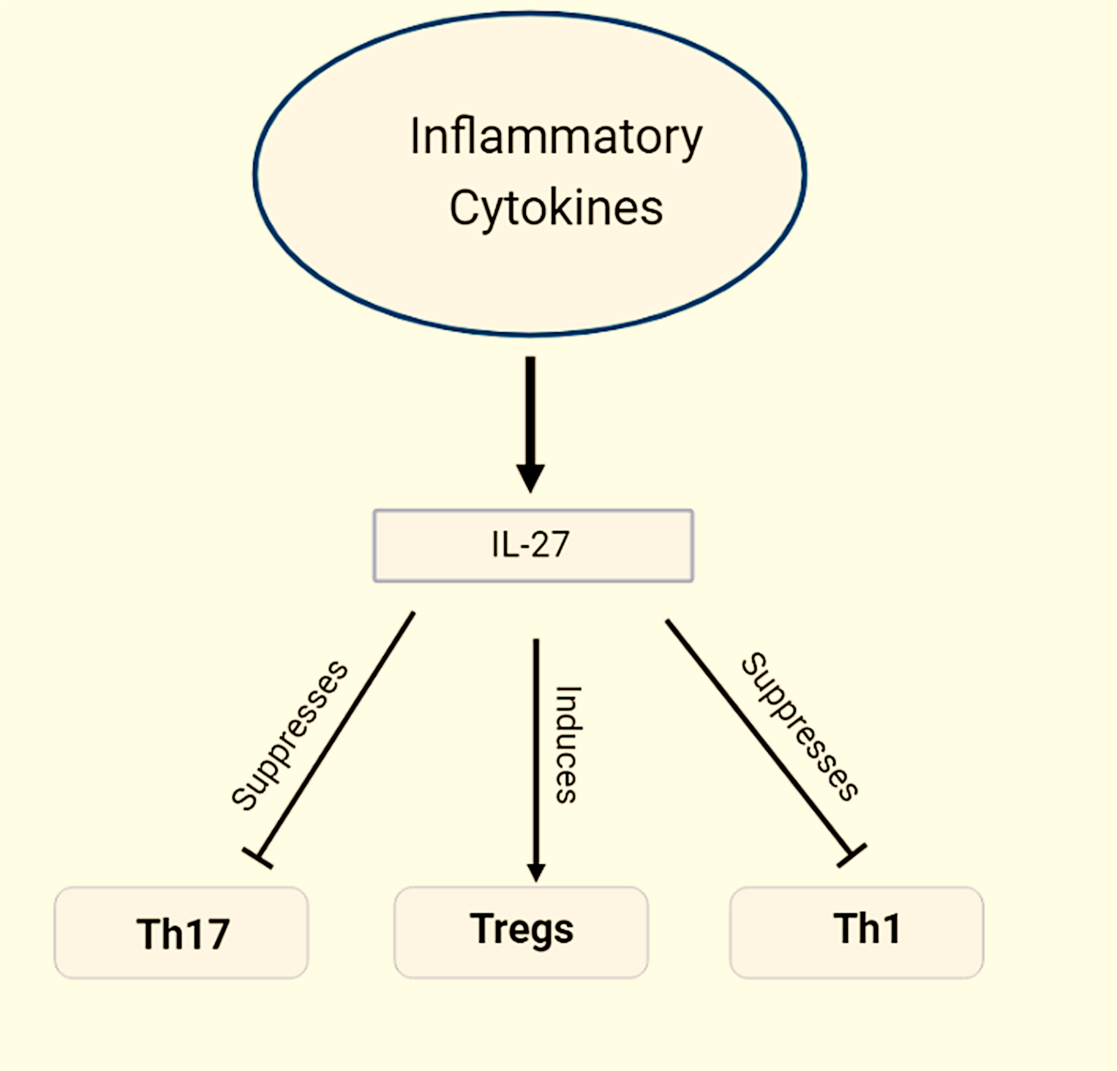
Involvement of interleukin 27 with Th-17, Tregs and Th1 [27].

IL-27 plays a crucial role in cardiovascular diseases (CVD), such as atherosclerosis, by primarily targeting inflammation. Its main mechanism involves modulating the differentiation of immune cells called Th17 cells. These Th17 cells contribute significantly to inflammation, but when IL-27 is present, it inhibits their ability to differentiate effectively. Consequently, this regulation of Th17 cell differentiation serves as a key mechanism by which IL-27 manages CVD. By interfering with the differentiation of Th17 cells, IL-27 helps to control the inflammatory response, ultimately influencing the progression of conditions like atherosclerosis. Th17 cells are a specific subset of immune cells that can undergo differentiation when regulated by IL-27. The Th17 cells have a recognized function in autoimmune and inflammatory conditions. In the subsequent processes, IL-27 has the capacity to affect the specialization of Th17 cells. It has been identified that IL-27 inhibits the proliferation and functioning of Th17 cells (Fig. **2**). The molecules known as interleukin-17 (IL-17) and interleukin-22 (IL-22) play a significant role in the creation of Th17 cells, which may undergo diminished production. By doing this, IL-27 assists in reducing the inflammatory response caused by Th17 cells [24]. IL-27 can encourage the development of regulatory T cells (Tregs), which are crucial for preserving immunological balance and controlling excessive inflammation. Th17 cells and other immune cells are kept in check by Tregs, which keeps inflammation at bay. IL-27 indirectly prevents the differentiation of Th17 cells by promoting Treg formation [25]. IL-27 possesses the capability to influence the signaling systems that govern the formation of Th17 cells. The development of Th17 cells relies on the stimulation of a protein called signal transducer and activator of transcription 3 (STAT3). IL-27 assists in diminishing the formation of Th17 cells and the accompanying inflammatory outcomes by impeding the activation of STAT3 (Fig. **2**) [26, 27].

Through several research studies, the effect of IL 27 on atherosclerosis was examined, and it was discovered that the CAD group’s blood IL 27 level was much higher than that of the groups without CAD (Table **1**) [28].

The identification of heightened levels of IL-27 in individuals with CAD leads to the prospect that IL-27 could play a part in the progression of the ailment. This finding indicates that the inflammatory and immune processes involved in atherosclerosis could lead to an enhancement of IL-27 production or discharge [29]. IL-27 controls immunological reactions, Solve R2, 8, such as the development of immune cells like Th17 cells. Since IL-27 levels are higher in CAD patients, these factors may be affecting immune cell development and function, which may have an impact on the immune response within atherosclerotic plaques [30]. In a case-control study examining 120 patients diagnosed with ischemic heart disease (IHD), including 60 with AMI and 60 with unstable angina (UA) R2(9), alongside a control group of 60 healthy patients, it was found that elevated serum levels of IL-27 were linked to IHD. The study highlights the role of IL-27 in the pathogenesis of ischemic heart disease. Elevated IL-27 levels in patients with acute myocardial infarction or unstable angina (UA) R2(9) suggest its involvement in myocardial damage. IL-27 affects the balance between Th1 and Th2 cells, proinflammatory cytokine production, and regulatory T cell responses, potentially contributing to the development and progression of IHD. Furthermore, IL-27 may be linked to infectious agents associated with cardiovascular events. These findings underscore the potential of IL-27 as a therapeutic target and the need for further research to explore its predictive and prognostic value in cardiovascular disease [31].

#### 5.1.1 Impact of IL-27 on NLRP3 Inflammasome Activation in Atherosclerosis

The most common and significant pathological state in atherosclerotic vascular disorders is atherosclerosis, which is the root cause of many cardiovascular diseases (CVDs) [32]. Multiple studies have clarified the relationship between atherosclerosis and the activation of NLRPs inflammasome [32-34]. One of the subgroups of PRPs is known as nucleotide-binding oligomerization domain (NOD)-like receptors (NLRs). Pyroptosis is brought on by activating the NLRP3 inflammasome, which results in caspase-1 maturation and the production of inflammatory cytokines such as IL-18 and interleukin-1β (IL-1β). It has recently been established that the excessive activation of the NLRP3 inflammasome mediates inflammatory responses and has a role in the development and initiation of atherosclerosis [33]. From a therapeutic perspective, blocking the NLRPs inflammasome can effectively suppress atherosclerosis. New treatment options for atherosclerosis prevention are available when one moves upstream in the inflammatory cascade from CRP to IL-6 to IL-1. These options center on the primary IL-6 signaling pathway and, ultimately, on inhibiting the NLRP3 inflammasome, which produces IL-1β [35]. In macrophages, Th1 cells, and human peripheral blood mononuclear cells (PBMCs), IL-27 significantly promotes inflammation. By triggering LPS-ATP signaling *via* both NLRP3-dependent and independent pathways, it raises the level of IL-1β. IL-27 functions as a priming signal, increasing pro-IL-1β production induced by LPS to enhance NLRP3 inflammasome activation. It also serves as a secondary signal through enhancing the induction of IL-1β, ATP effects, and the activity of caspase-1. Moreover, TLR4 can be activated by IL-27, which then causes relocalization of CD14-TLR4. This triggers the STAT3-nuclear factor-kappa B pathway, which in turn mediates the LPS response in monocytes [28]. It would be advantageous to target the NLRP3 inflammasome in order to stop atherosclerosis from progressing. Furthermore, because of its correlation with LPS-induced inflammation, IL-27 may also function as a possible marker or indication for atherosclerosis [28]. The objective of the CANTOS study was to assess the effectiveness of canakinumab, an inhibitor of the IL-1β to IL-6 to CRP axis, in mitigating cardiovascular events among patients who recently suffered a myocardial infarction. This study confirmed the inflammatory hypothesis regarding atherothrombosis by showing a decrease in serious cardiovascular events after canakinumab therapy. It proved to be effective in reducing the relative risk of hard cardiovascular events, such as myocardial infarction, stroke, or cardiovascular mortality, by 20% [35].

#### 5.1.2 IL-27 in CAD

According to a study, IL-27 was shown to be elevated in coronary artery disease (CAD), especially in patients with acute coronary syndrome (ACS). This implies that IL-27 might be involved in the onset of CAD. Furthermore, a noteworthy correlation was observed between IL-27 levels and the extent of coronary artery stenosis, as indicated by the Genisini score. According to this, IL-27 may promote Th1 cell differentiation and the release of associated cytokines, which may accelerate the development of CAD [4].

### 5.2 IL‐28A, IL‐28B, and IL‐29

**Fig. (3):**
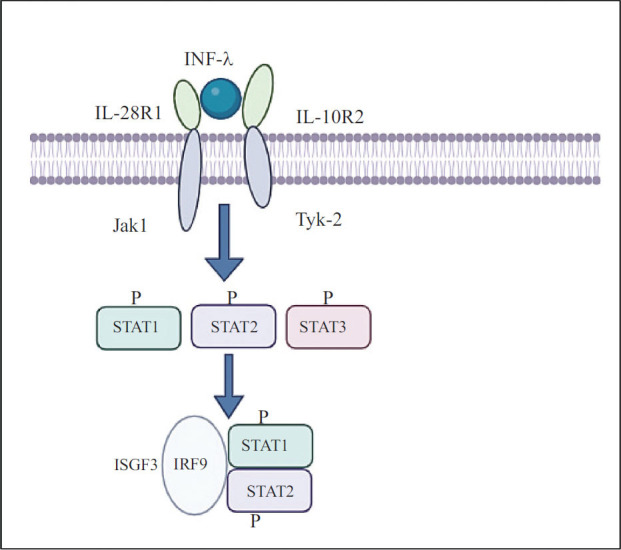
IFN λ induces activation of the Jak/STAT signaling pathway.

A new family of cytokines called IFN λ1 (IL-29), IFN λ2 (IL-28A), and IFN λ3 (IL-28B) resemble type I interferons (IFN) [6]. Type-I interferon (IFN), which also consists of the cytokines that are similar to IL-10 (IL-10, IL-19, IL-20, IL-22, IL-24, and IL-26) [36], demonstrates a connection between the IL-10 family and these cytokines [6, 37, 38]. Interleukin-28/29 (IL- 28/29) is a designation utilized to define two novel signaling molecules that have been recently discovered in the human genome: interleukin-28 (lL-28A, IL-28B) and IL-29 [39]. Their similar genes and activation of the same signaling pathway (JAK-STAT) result in antiviral, antiproliferative, and *in vivo* anticancer actions. Despite these similarities, IFNs λ differ from type I IFNs in binding to a distinct receptor of IFNLR1 and IL10R2. Their categorization as type III IFNs resulted from this difference. They share biological roles, signaling pathways, and induction processes with type I IFNs despite their structural as well as genetic differences [6]. IL-28 and IL-29, which possess antiviral properties, are produced in a similar manner to interferons in vitro in response to viral infections. Because of the ability of STAT, IRF, and ISGF activation through the IL-28 receptor to cause a cellular state that hinders the development of viruses in a controlled environment, all three cytokines are categorized as interferons [6, 38, 39]. The incorporation of IL-28 as an immune booster in vaccinations for small animals has been demonstrated to enhance the generation of interferon-gamma that specifically targets the antigen. This also strengthens the ability of CD8+ T cells to destroy harmful cells, thus reaffirming the significance of IL-28 in the body's adaptive immune system [40]. Cardiovascular disease (CVD) now accounts for more deaths worldwide than any other condition. This pandemic might have a substantial social and economic impact on developing countries [41]. Inflammation has a big impact on CVD, and signs of inflammation can predict when the disease will strike again [42]. The regulation of inflammation can largely be managed by the group of cytokines called interleukin (IL)-10. This group includes IL-10, IL-19, IL-20, IL-22, IL-24, IL-26, as well as the less closely related IL-28A, IL-28B, and IL-29 [43]. The advancement of new treatment approaches could arise from the control of inflammation's significant association with the onset and progression of cardiovascular disease (CVD) [10]. Atherosclerosis, heart attack, high blood pressure, weakened heart function, irregular heartbeat, heart muscle disease, and associated heart conditions constitute the majority of cardiovascular disease (CVD) [44]. Cardiovascular disease (CVD) is believed to be closely linked to inflammatory diseases, which are caused by various cytokines and their receptors [45]. Interleukin 10 (IL-10) is an important anti-inflammatory cytokine produced by both Th2 cells and macrophages. It effectively deactivates both macrophages and T cells [46]. The theory suggesting that IL-10 has a role in arterial thrombotic diseases is reinforced by epidemiological investigations that have found a link between reduced levels of IL-10 in the bloodstream and a heightened chance of various cardiovascular disease (CVD) outcomes, such as acute coronary syndrome (ACS) and ischemic stroke (IS) [47, 48]. The recently discovered additions to the IL-10 family, namely IL-28A, IL-28B, and IL-29, were derived from the genetic information present in the human genome. These three cytokines are linked to the category of type I IFN group and the IL-10 group. There has been limited research conducted on the associations between these three cytokines and cardiovascular ailments [49]. Further examination is necessary to determine the precise impact of IL-28a, IL-28b, and IL-29 on cardiovascular ailment. Interleukin-29 (IL-29) has the ability to trigger many signaling pathways, such as the nuclear factor-kappa B (NF-κB), mitogen-activated protein kinase (MAPK), protein kinase B (Akt), and Janus kinase/signal transducer and activator of transcription (JAK-STAT) pathways. This activation impacts cellular processes in various cell types and causes the production of inflammatory components (Fig. **3**) [50].

The activation of the JAK-STAT pathway is triggered when a ligand, such as cytokines or hormones, attaches to its particular receptor present on the cell membrane, causing receptor dimerization. By trans-phosphorylation, this dimerization initiates the activation of Janus kinases (JAKs) linked to the receptor. The receptor's particular tyrosine residues are subsequently phosphorylated by the phosphorylated JAKs, forming binding sites for latent cytoplasmic STAT proteins. JAKs then phosphorylate these STAT proteins, forming dimers that eventually go into the nucleus. When STAT dimers enter the nucleus, they attach to particular DNA sequences and encourage the transcription of genes that are involved in a number of different cellular functions, including differentiation, death, and proliferation. Suppressors of cytokine signaling and protein tyrosine phosphatases, which aid in preventing the signaling cascade, are examples of inhibitory molecules that control this pathway. Among other things, this cascade contributes to the formation of vascular lesions and blood vessel constriction, which may lead to cardiovascular disease components [50].

### 5.3 Interleukin‐31

Members of the IL-6 family are strongly associated with cardiovascular disorders like hypertension, atherosclerosis, aortic dissection, fibrosis of the heart, and cardiomyopathy, according to preclinical and clinical data [51]. A component of the IL-6 cytokine group is interleukin-31 (IL-31), which belongs to the family of cytokines with a structure composed of four-helix bundles. IL-31 is primarily produced by CD4+ T helper 2 (TH2) cells and CD45RO+ T cells associated with the skin, although human dendritic cells also produce smaller quantities of IL-31. R1(1) IL-31 production is induced by interleukin-4 in TH2 cells, and its expression is triggered by staphylococcal enterotoxin B in peripheral blood mononuclear cells [52]. However, the upstream signaling pathway responsible for the creation of IL-31 remains largely unknown [53, 54].

R1(1) IL-31 is part of the IL-6 cytokine family and is characterized by its four-helix bundle structure, a feature typical of cytokines in this family. This structure enables its interaction with specific receptors, leading to downstream effects in immune response regulation.

IL-31 exerts its effects by interacting with a receptor complex composed of IL-31RA and OSMR. Once bound, it activates several intracellular signaling pathways, including STAT1, STAT3, and STAT5, which influence gene expression and cellular responses. IL-31 also regulates the MAPK and PI3K pathways, which are critical for processes such as proliferation, differentiation, apoptosis, and survival. R1(1) Blocking IL-31 downstream signaling could be a useful treatment for several inflammatory conditions, including prurigo [53]. IL-31 is now understood to play a role in various illnesses, including atopic dermatitis, bowel inflammation, and cutaneous lymphomas. Despite its established role in these conditions, there is insufficient evidence to support its direct involvement in cardiovascular diseases [51]. There is increasing evidence that indicates inflammation plays a significant role in the development of cardiovascular disease. Members of the IL-6 family regulate immune responses and inflammatory actions, which in turn lead to the manifestation of cardiovascular ailments [51]. Both inflammatory and non-inflammatory impacts of IL-6 can be attributed to its production by cardiovascular cells such as endothelial cells, vascular smooth muscle cells, ischemic myocytes, along with immune cells like macrophages and monocytes. Due to IL-6's involvement in both inflammation and heart metabolism regulation, it has drawn significant attention from the medical community [55].

Although IL-31 belongs to the IL-6 family, its role in cardiovascular diseases is not well understood. One factor influencing IL-31's diverse effects is its interaction with various signaling pathways, including the activation of STAT1, STAT3, and STAT5, which regulate gene expression. Moreover, it influences the MAPK and PI3K pathways, which are critical for cell proliferation, differentiation, and survival. In vitro studies show that IL-31 inhibits Th17 cell development while promoting Th1 cells [56]. While direct evidence of IL-31's role in cardiovascular disease is limited, there is growing interest in understanding its potential mechanisms in these conditions. IL-31 may influence the inflammatory processes involved in atherosclerosis and hypertension by interacting with endothelial cells and vascular smooth muscle cells. By regulating STAT3 and MAPK signaling pathways, IL-31 might contribute to vascular remodeling and inflammation, which are key factors in cardiovascular disease progression. IL-31's ability to modulate Th1 and Th17 immune responses suggests it may play a role in chronic inflammation observed in cardiovascular disorders R1(1).

### 5.4 Interleukin‐32

IL-32 is a protein messenger that triggers an immune response and is generated by stimulated natural killer cells, T lymphocytes, cells lining the surface of organs, circulating immune cells, supportive tissue cells, connective tissue cells, and liver cells [51, 52]. It has seven isoforms IL-32α, IL-32β, IL-32γ, IL-32δ, IL-32ε, IL-32ζ, IL-32θ and nine exons [53, 54]. IL-32α R2(10), IL-32β, IL-32γ, IL-32δ isoforms were noticed in the NK cells which were stimulated by IL-2; IL-32ε, IL-32ζ expressed in the activated T cells [53, 55]. IL-32θ was noticed in the dendritic cells [53, 56]. IL-32γ consists of the longest amino acid chains [53]. IL-32γ gives most of the pro-inflammatory reactions [57]. The specific cell surface receptor that IL32 requires to attach or to function is still unknown. But some receptors are found like proteinase3 and integrins, aVb3 and aVb6 [57, 58]. Proteinase3 has a high tendency to bind with IL-32 [53]. It consists of different characteristics of proinflammatory cytokines. It turns on TFNα, IL-1β, IL-6, chemokines, and IL-8 by NF-κB and p38 mitogen-activated protein kinase activation [52, 59, 60]. IL-32 increases itself by IL-1β, and IL-6 production, which is induced by muramyl dipeptide as NOD1 and NOD2 (nucleotide oligomerization) domains. It uses the caspase-1 mechanism [52, 60]. TFNα, IL-1β, IL-6, and IL-8 are responsible for atherogenesis; especially TFNα is related to CVD and atherogenesis; IL-6 is related to thrombocytosis; IL-8 related with plaque formation; IL-1β related with vascular inflammation and correlate with atherogenesis. It is found that lowering the IL-32 level reduces pro-inflammatory cytokines and procoagulant effects. IL-32 expression is also found in arterial vessel walls [57]. IL-1β once again stimulates the production of IL-32 in cells lining blood vessels and various types of cancer cells. Elevated concentrations of IL-32 have been observed in the serum levels of various illnesses. IL-32 is expressed in endothelial cells and its activation is facilitated by Akt (a protein kinase B) [58]. Akt plays a vital role in transcription, programmed cell death (apoptosis), cellular reproduction (proliferation), and the regulation of glucose metabolism [61, 62]. Additionally, the presence of IL-32 expression can once again be observed in macrophages, which are readily present in atherosclerotic lesions. Upon being activated by cytokines like interferon-gamma (IFNγ) and a Toll-like receptor (TLR)3 ligand Poly I: C, macrophages exhibit a substantial increase in both mRNA and protein levels of IL-32. Research has indicated their active participation in the development of atherosclerosis [57, 63]. Moreover, IL-32 consists of SNP [64]. Promoter SNP (single nucleotide polymorphism) in IL-32 linked with high HDLC level. SNP also affects the isoforms in PBMCs (peripheral blood mononuclear cells). Lower levels of IL-32β were found in RA patients with these types of SNP. After stimulation, it also reduces cytokine production of PBMCs [57, 65]. This type of mutation in the IL-32 gene may give benefit in lowering CVD risks [57]. Besides that, IL-32 activates the NOD2/ NOX2 pathway and interrupts normal cardiomyocyte function. Mature types of NOX2 have developing expression during tissue ischemia and they are related to coronary artery patients [66].

#### 5.4.1 IL-32 Role on Arterial Stiffening

Arterial stiffening refers to reduced flexibility as a result of alterations in the artery wall's characteristics, especially in the medial layer. The arteries' capacity to absorb the pulsatile force of blood expelled from the heart is impacted by this weakened distensibility [67]. The onset of heart failure and cardiovascular diseases (CVD) is greatly influenced by arterial stiffness, which is an important factor in cardiovascular health [67]. It is a crucial factor in determining cardiovascular risk because it can cause vascular blockage, which can result in ischemia, a diminished flow of blood to limbs or organs. In particular, aortic stiffness, which tends to worsen with age, increases pulse pressure and raises the risk of cardiovascular disease [68]. The study found a strong correlation between arterial stiffness and IL-32 expression, suggesting that IL-32 may affect the general health of blood vessels by regulating the suppleness and elasticity of arterial walls. More specifically, lower cumulated lateral translation (CLT-CCA), a measure of decreased arterial stiffness, was associated with higher IL-32 isoform levels. Both the control group and people living with HIV (PLWH) showed this association, especially in those that had greater CLT-CCA values. Notably, among PLWH, the significant connection predominantly identified in the upper quartiles of CLT-CCA measurements indicates that the negative link between IL-32 isoforms and arterial stiffness was most evident in people with less progressed vascular disease. But when age was taken into account, this relationship was mostly seen in comparatively younger PLWH, indicating that IL-32 may hasten the beginning of vascular illness in this population at a younger age [68].

### 5.5 Interleukin‐33

**Fig. (4):**
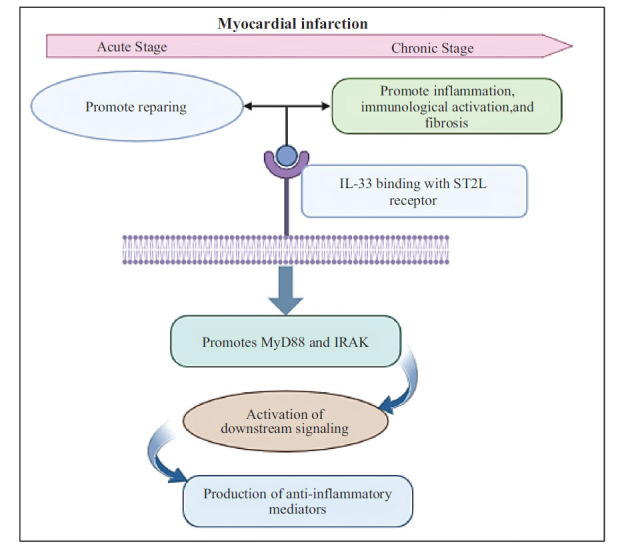
IL-33/ST2L signaling pathway and impact on myocardial infarction.

The cytokine interleukin-33 is a recent addition to the IL-1 family [69-74]. The nuclear factor known as IL-33 was first discovered in 2003 and was thought to originate from high endothelial venules. After a span of two years, Schmitz and associates recognized IL-33 as a constituent of the IL-1 family and also confirmed its function as a ligand for the ST2 receptor (often referred to as IL-1RL1). It is principally generated by endothelial cells, epithelial cells, and fibroblasts [75]. Through its binding to ST2L on the membranes of inflammatory cells, MAPK-kinases and other metabolic pathways are activated. As a result of these interactions, the inhibitor of the nuclear factor-kappa B (IKK) complex is activated, allowing NF-kappa B to become active and exercise its proinflammatory effects. Because of its ability to bind IL-33, sST2 seems to play the role of a decoy receptor, preventing the presence of this molecule from interfering with the interaction between ST2L and ST2s. When sST2 interacts with IL-33, it has the potential to decrease NF-κB synthesis and activation, hence dampening the inflammatory response. The decoy receptor function of sST2 dampens the beneficial effects of IL-33 [76]. Recent studies indicate that IL-33 and ST2L play a defensive role in atherosclerosis and cardiac restructuring. At a biological level, IL-33 is mostly absent in blood-forming cells but widely present in supporting cells like connective tissue cells, muscle cells, cells lining blood vessels, and cells forming the outer layer of organs. Atherosclerotic plaques showed a decrease in the number of F4/80+ macrophages and CD3+ T lymphocytes following IL-33 therapy. Moreover, the protective effects against atherosclerosis of IL-33 were hindered by combining it with a neutralizing anti-IL-5 antibody, indicating that the assistance provided by IL-33 *in vivo* is possibly mediated through an IL-5-driven response. One possible additional way IL-33 reduced atherosclerosis is by helping to prevent the creation of macrophage foam cells through its action. It was also demonstrated that IL-33, a substance that counters the effects of angiotensin II and phenylephrine and causes heart muscle cell enlargement, is a protein produced by heart fibroblasts in response to biomechanical stimuli. Moreover, the introduction of IL-33 *in vivo* helped diminish hypertrophy and fibrosis induced by TAC by inhibiting the activation of NF-κB. In conclusion, sST2 serves as a soluble imitation receptor in the heart muscle, obstructing the anti-enlargement effects of IL-33. By restricting caspase-3 function and boosting the generation of the 'inhibitor of cell death' (IAP) group of proteins, IL-33 can potentially diminish the demise of heart muscle cells, lessen heart attack damage and scarring, and improve the pumping ability of the ventricle in a living organism. Even though IL-33 is widely recognized as a necessary protein for Th2-mediated protection of the body, recent findings indicate that the IL-33/ST2 pathway also has an important role in the cardiovascular system. IL-33, found in the blood vessels, has been linked to offering numerous safeguards against atherosclerosis and heart fibrosis. Moreover, studies indicate that sST2 might serve as a biomarker for foreseeing death in various heart-related illnesses. Hence, adjusting the IL-33/ST2 pathway could serve as a beneficial novel technique for managing and averting heart-related ailments [69, 77-84]. Kidney transplant patients' exposure to cardiovascular risk is estimated using the Comprehensive Registry of Cardiovascular Risk Events (CRCRTR-MACE) (RTR). UVA analysis was used to compare interleukin levels between those with low and high CRCRTR-MACE scores. The only interleukin substantially linked to a high CRCRTR-MACE score was IL-33 [82]. Increasing soluble ST2 levels in the blood of mice after myocardial infarction has brought attention to a putative link between IL33 and cardiovascular disease. After a person has a myocardial infarction, the concentration of soluble ST2 rises and is associated with reduced left ventricular function and a bad result, indicating that soluble ST2 may be used as a predictor of death or heart failure. The fact that IL-33 treatment has a protective impact on cardiac overload suggests that soluble ST2 has a physiological role to play since this effect is mediated by a decrease in cardiomyocyte hypertrophy and ultimately results in less cardiac dysfunction and enhanced survival [83]. Consequently, an increase in sST2 might have a harmful predictive effect on cardiovascular risk characteristics and could hinder the protective effect of IL-33 on heart cells. The functioning of the heart muscle may worsen due to a decrease in the heart-protective benefits of IL-33. This occurs through the ST2/IL-33 pathway. Heart attack, programmed cell death of heart muscle cells, activation of inflammation in the heart, scarring, and adverse changes in heart tissue appear to be reduced by this. Individuals with heart attack, heart failure, and difficulty breathing may find it advantageous to utilize sST2 as a medical indicator for predicting outcomes in the process of categorizing risk by means of analyzing blood samples. The routine examination of a patient with a heart attack and heart failure should include the evaluation of sST2 levels. However, more research is necessary to better emphasize the proof for this procedure [76]. The study on a cohort of 191 people with advanced systolic congestive heart failure found IL-33's antioxidant effects on heart failure patients. Contrary to expectations, IL-33 is unexpectedly linked with higher levels of oxidative damage indicators rather than a decrease in oxidative stress. Nevertheless, studies on cardiomyocytes demonstrated that IL-33 might increase the activity of antioxidant enzymes and decrease oxidative stress; however, sST2 prevented this effect, indicating a complicated interplay between these biomarkers in heart failure [84]. Upon binding to a receptor complex comprising ST2 and IL-1RAP, IL-33 promotes MyD88 along with IRAK to be recruited. This activation triggers downstream signaling pathways that result in the generation of anti-inflammatory cytokines, such as MAP kinases. In endotoxemia, proinflammatory cytokine production and TLR-4 gene expression are suppressed, which allows IL-33 signaling to increase inflammation [75]. The protective benefits of IL-33/ST2L signaling can be countered by the presence of soluble ST2 (sST2), which functions as a decoy receptor. This interference may accelerate the development of heart disease by causing the heart to become increasingly inflamed, and severely altered (Table **2**) [85]. Another study confirmed that, in the acute phase of myocardial infarction, IL-33 both inhibits the Th1 immune response and promotes heart healing. In contrast, IL-33's binding with the ST2L receptor during the chronic phase promotes fibrosis, immunological activation, along with eosinophilic inflammation (Fig. **4**) [86].

### 5.6 Interleukin-38

Within the IL-1 family cluster lies the unique IL-1-like gene, IL-38. On the same DNA strand as IL-1RN, the IL-38 gene is situated at 49,479 base pairs upstream. Due to its amino acid resemblance to the naturally occurring IL-1Ra and the fact that IL-38 could bind to the soluble IL-1 receptor type I (IL-1RI), it was hypothesised in 2001 that IL-38 functioned as an IL-1 receptor antagonist. It was previously believed that IL-1RI was an IL-38 receptor [21, 87, 88]. When IL-38 binds to a particular receptor, it inhibits signalling pathways associated with inflammation in inflammatory diseases, such as nuclear factor kappa B (NK-κB) and mitogen-activated protein kinase (MAPK). Additional studies have demonstrated that IL-38 possesses anti-atherosclerotic properties as well, which lower the likelihood and frequency of cardiovascular events. In order to prevent inflammation, lessen abnormal neovascularisation, and prevent apoptosis, IL-38 can, on the one hand, control both innate and adaptive immunity [89, 90]. By inhibiting the activation of the NOD-like receptor thermal protein domain-associated protein 3 (NLRP3) inflammasome, promoting the differentiation of M1 macrophages into M2 macrophages, and increasing the secretion of anti-inflammatory cytokines like IL-10 and transforming growth factor-β, IL-38 can relieve myocardial ischemia-reperfusion injury [91].

### 5.7 Interleukin-11

IL-11, IL-6, IL-31, LIF, OSM, CNTF, CT-1, cardiotrophin-2 (CT-2) and cardiotrophin-like cytokine (CLC) are all members of the IL-6 family [92]. According to earlier research, IL-11 is thought to be a major factor in cardiovascular illnesses [92]. The extracellular signal-regulated kinase (ERK1/2)/interleukin-11 (IL-11) signalling pathway is markedly activated by Ang II, according to recent research. Heart failure has been linked to persistent activation of the IL-11 signalling pathway, albeit the precise mechanism is uncertain. Consequently, it may be clinically advantageous to negatively regulate these signalling pathways in order to cure hypertensive heart failure [93]. The STAT3 pathway may be activated by IL-11, which has been shown to target cardiac myocytes and potentially prevent hydrogen peroxide-induced cell death [92]. IL-11 mainly stimulates angiogenesis in endothelial cells, which helps prevent coronary heart disease. By triggering fibroblast activation and smooth muscle cell transformation, IL-11 also encourages vascular remodelling. Through the non-classical ERK pathway, IL-11 may contribute to the development and progression of pulmonary hypertension through the JAK/STAT3 pathway and the processes leading to aortic dissection [94].

## CONCLUTION & FUTURE PRESPECTIVES

In conclusion, members of the interleukin family are critical in the emergence of CVDs such as atherosclerosis, cardiac fibrosis, hypertension, and cardiac hypertrophy. It is apparent that these cytokines exploit the intricate networks to influence the development of heart disease. These cytokines can play a variety of functions in the same disorders, most likely due to various routes, receptors, or levels, despite their non-critical roles. To develop therapeutic targets for cardiovascular disorders, more studies are necessary. Nevertheless, these studies provide us with useful treatment approaches and suggestions for further research. Modifying the production of interleukin or its effects might provide opportunities for developing innovative treatment techniques. Furthermore, interleukin levels (IL-27, IL-28, IL-29, IL-31, IL-32, and IL-33) may enhance the treatment approach if the temporal dynamics of those levels are investigated prior to or at the onset of cardiovascular events [101-103]. Understanding the roles played by these different systems in the context of a disease is essential. The cardiovascular system is made up of several tissues arranged in a unique way. The way these cell types interact is crucial for both preserving health and influencing the course of illness. Treatments that target interleukins, such as IL-27, IL-28, IL-29, IL-31, IL-32, and IL-33, have shown promise in reducing fibrosis and inflammation, two important processes linked to the development of heart disorders such as atherosclerosis and heart failure. Recent data indicates that interleukins have a major effect on the signaling pathways in the cardiovascular system. Moreover, greater investigation is needed into interleukins as possible targets for novel therapeutic strategies. More research in these areas is urgently needed. Future research, especially through in vitro models and clinical trials, should explore the precise immunomodulatory effects of interleukins on heart disease.

## Figures and Tables

**Table 1:** Examining the serum level of IL-27 of patients with various specifications.

**Population**	**Finding (Serum Level of IL- 27 Compared to the Control Group)**
264 hospitalized patients with chest pain who had coronary angiography	Higher
60 patients with AMI	Higher
60 patients with UAP	Higher
60 healthy controls	No significant difference
43 SAP patients	No significant difference
47 chest pain syndrome patients as a control group	Weak negative correlation with LVEF in CAD patient

**Table 2:** Drugs that inhibit the function of interleukin.

**Drug**	**Type**	**Mechanism of Action**	**Result**	**References**
Canakinumab	IL-1β antibody	Inhibit IL-1β interaction with IL-1RI, IL-1RII receptors.	Decreased occurrence of ischemic events, Lower rate of hospitalization due to heart failure, Increase in infection-related deaths, Reduction in occurrences of rheumatologic diseases.	[95]
Tozorakimab	IL-33 antibody	Bind and block IL-33ox signaling *via* the RAGE/EGFR pathway.	Reduce inflammation along with epithelial remodeling.	[96]
REGN3500 (Itepekimab)	IL-33 antibody	Selectively target IL-33, inhibit the interaction between IL-33 and its receptors, and disrupt subsequent signaling events pathway.	Effective in treating asthma and COPD.	[97, 98]
Etokimab	IL-33 antibody	Under clinical investigation.	Decrease inflammation, effective in treating COPD and asthma.	[99, 100]

## LIST OF ABBREVIATIONS

ACS: Acute Coronary Syndrome
CAD: Coronary Artery Disease
CHIP: Clonal Hematopoiesis of Undetermined Potential
COPD: Chronic Obstructive Pulmonary Disease
CVD: Cardiovascular Diseases
IL: Interleukin
MAPK: Mitogen-activated Protein Kinase
NF-κB: Nuclear Factor-kappa B
